# Laparoscopic Adjustable Gastric Banding Connecting Tube Causing Small Bowel Obstruction and Perforation

**DOI:** 10.1155/2013/296037

**Published:** 2013-12-04

**Authors:** Mojtaba Hashemzadeh, Mahmoud KaramiRad, Leila Zahedi-Shoolami

**Affiliations:** Private Bariatric Clinic, No. 204, Nader Building, 47th Avenue, Yousefabad, Tehran, Iran

## Abstract

*Background*. Laparoscopic adjustable gastric banding (LAGB) is an effective method of reducing excess weight in obese patients. We report a patient who developed a bowel obstruction caused by the connecting tube between the gastric band and the injection port. *Case Presentation*. The patient was a 42-year-old Caucasian female who had undergone LAGB 19 months earlier. She presented with dehydration, low-grade fever, tachycardia, and mild abdominal tenderness. Laparotomy revealed that the connecting tube was looped around the mesentery, and a loop of small bowel was incarcerated between the tube and the mesentery. The incarcerated small bowel loop was perforated in two places. *Conclusion*. Surgeons should be aware of the possibility of obstruction caused by the connecting tube in patients who have undergone LAGB.

## 1. Background

Obesity is a major problem that affects a large number of people worldwide. Laparoscopic adjustable gastric banding (LAGB) is an effective method of reducing the excess weight in obese patients. This procedure is favored by both patients and surgeons because of its advantages in terms of short hospital stay, low mortality rate, effective loss of excess weight, and improvement in comorbid conditions [1, 2].

The overall early and late complication rate is estimated to be between 2.2% and 20% [2, 3].

Obstructions caused by the connecting tube between the gastric band and the injection port are uncommon but are potentially fatal [4–7]. We report a patient who had undergone LAGB 19 months earlier, who presented with several episodes of vomiting, mild abdominal pain, and a low-grade fever. 

## 2. Case Presentation

A 42-year-old woman presented to our clinic with a 3-day history of severe vomiting, abdominal pain, and a low-grade fever. She had undergone LAGB 19 months previously. Her body mass index was 35.5 kg/m^2^ at the time of LAGB and 27.5 kg/m^2^ at the time of the current presentation. 

Physical examination showed dehydration with an oral temperature of 37.9°C and a heart rate of 108 beats/minute. She had mild generalized abdominal tenderness without distension, guarding, or rebound. Laboratory tests showed a white blood cell count of 4800 cells/*μ*L without a shift to the left. 

The patient had a barium meal X-ray that showed multiple air-fluid levels and dilated intestinal loops, indicating partial bowel obstruction. Ultrasonography showed dilated bowel loops and free intra-abdominal fluid. She was transferred to the operating room with an initial diagnosis of partial bowel obstruction.

Laparotomy was performed through an upper midline incision. The connecting tube between the gastric band and the injection port was found to be looped around the mesentery, and a loop of small bowel was incarcerated between the connecting tube and the mesentery. At 60–70 cm from the ileocecal valve, the bowel was adherent to the connecting tube in three distinct places, causing an obstructed loop. The small bowel proximal to the obstruction was dilated, and there was a gastric perforation adjacent to the band. There were free pus and fecal material in the abdominal cavity.

**Figure 1:**
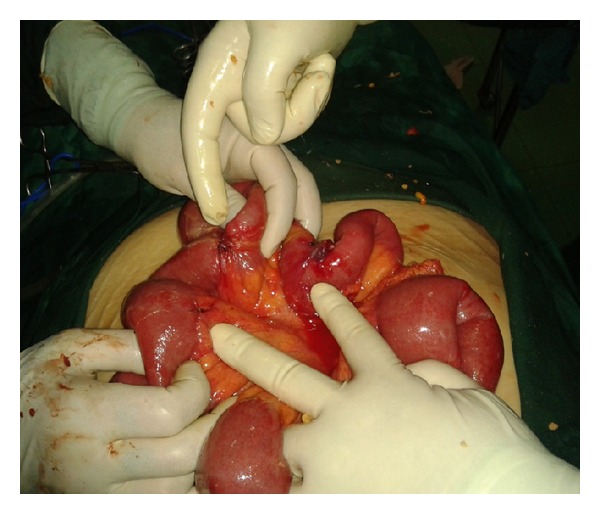
Small bowel perforations.

After dividing the adhesions and freeing the connecting tube, a thorough examination revealed two perforations of the small bowel (Figure 1), which were repaired using an omental patch. A Penrose drain was placed in the left lower quadrant. 

The gastric band was removed and the abdominal cavity was lavaged with about 6 L of normal saline.

The patient underwent a Gastrografin study on postoperative day 7, which did not show any leakage. She was then started on a fluid diet and was discharged 3 days later on postoperative day 10. 

## 3. Discussion

Although LAGB has many benefits, several complications have also been reported. The most common complications are pouch enlargement, band slippage, band erosion, port-site infection, and port breakage [2, 3, 8–13]. Some more serious and potentially life-threatening complications have also been reported. There have been a few reports of complications caused by the connecting tube, including perforations of the small bowel and colon [4–7, 14–16]. 

It is important to note that even though our patient had partial bowel obstruction with small bowel perforations, she did not look particularly unwell and did not have leukocytosis. The main features of her presentation that guided us towards performing an exploratory laparotomy were dehydration, tachycardia, and mild abdominal tenderness, together with her abdominal X-ray findings.

## 4. Conclusion

In patients who have undergone LAGB, the possibility of obstruction caused by the connecting tube should be considered. It is important to remember that physical findings may not be reliable in obese patients, which may increase the difficulty of accurate diagnosis and appropriate management. 

Considering that there are not many reports of complications associated with bariatric surgery procedures, we hope that this report will encourage our colleagues to share their experiences of complications, in order to reduce long-term mortality rates.

## Abbreviations

LAGB: Laparoscopic adjustable gastric banding.

## References

[1] Richardson J, Smith B (2012). Laparoscopic gastric banding. *Minerva Chirurgica*.

[2] Eid I, Birch DW, Sharma AM, Sherman V, Shahzeer K (2011). Complications associated with adjustable gastric banding for morbid obesity: a surgeon’s guide. *Canadian Journal of Surgery*.

[3] Alhamdani A, Wilson M, Jones T (2012). Laparoscopic adjustable gastric banding: a 10-year single-centre experience of 575 cases with weight loss following surgery. *Obesity Surgery*.

[4] Tan LBK, So JBY, Shabbir A (2012). Connection tubing causing small bowel obstruction and colonic erosion as a rare complication after laparoscopic gastric banding: a case report. *Journal of Medical Case Reports*.

[5] Zappa MA, Lattuada E, Mozzi E (2006). An unusual complication of gastric banding: recurrent small bowel obstruction caused by the connecting tube. *Obesity Surgery*.

[6] Van De Water W, Vogelaar FJ, Willems JM (2011). An unusual complication 4 years after laparoscopic adjustable banding: jejunal obstruction due to the connecting tube. *Obesity Surgery*.

[7] Agahi A, Harle R (2009). A serious but rare complication of laparoscopic adjustable gastric banding: bowel obstruction due to caecal volvulus. *Obesity Surgery*.

[8] Baraket O, El Ajmi M, Chouchene A (2010). Results of laparoscopic treatment of morbid obesity: about 27 cases. *Tunisie Medicale*.

[9] Martikainen T, Pirinen E, Alhava E (2004). Long-term results, late complications and quality of life in a series of adjustable gastric banding. *Obesity Surgery*.

[10] McBride CL, Kothari V (2011). Evolution of laparoscopic adjustable gastric banding. *Surgical Clinics of North America*.

[11] Nguyen NT, Hohmann S, Nguyen X-M, Elliott C, Masoomi H (2012). Outcome of laparoscopic adjustable gastric banding and prevalence of band revision and explantation at academic centers: 2007–2009. *Surgery for Obesity and Related Diseases*.

[12] Sarker S, Herold K, Creech S, Shayani V (2004). Early and late complications following laparoscopic adjustable gastric banding. *American Surgeon*.

[13] Zuegel NP, Lang RA, Hüttl TP (2012). Complications and outcome after laparoscopic bariatric surgery: LAGB versus LRYGB. *Langenbeck’s Archives of Surgery*.

[14] Strobos E, Antanavicius G, Josloff R (2009). Unusual complication: small bowel obstruction caused by tubing of gastric band. *Surgery for Obesity and Related Diseases*.

[15] Shipkov CD, Uchikov AP, Uchikova EH (2004). Small bowel obstruction by the silicone tube of the gastric band. *Obesity Surgery*.

[16] Fass G, Simoens C, Mendes da Costa P (2010). Diffuse abdominal and port site pain caused by the connecting tube in gastric banding. *Obesity Surgery*.

